# Significance of urinary fatty acid-binding protein 4 level as a possible biomarker for the identification of minimal change disease in patents with nephrotic-range proteinuria

**DOI:** 10.1186/s12882-020-02122-y

**Published:** 2020-11-03

**Authors:** Marenao Tanaka, Masato Furuhashi, Norihito Moniwa, Takuto Maeda, Hideki Takizawa, Megumi Matsumoto, Akiko Sakai, Yukimura Higashiura, Yufu Gocho, Masayuki Koyama, Yayoi Ogawa, Tetsuji Miura

**Affiliations:** 1 Department of Cardiovascular, Renal and Metabolic Medicine, Sapporo Medical University School of Medicine, S-1, W-16, Chuo-ku, Sapporo, 060-8543 Japan; 2 Department of Nephrology, Teine Keijinkai Hospital, Sapporo, Japan; 3 Hokkaido Renal Pathology Center, Sapporo, Japan

**Keywords:** Fatty acid-binding protein, Nephrotic syndrome, Membranous nephropathy, Minor glomerular abnormalities, Kidney biopsy

## Abstract

**Background:**

Fatty acid-binding protein 4 (FABP4), but not FABP1 (liver-type FABP), is ectopically induced in injured glomerular endothelial cells, and urinary FABP4 (U-FABP4) level is associated with proteinuria and renal dysfunction in a general population.

**Methods:**

The clinical significance of U-FABP4 was investigated in 81 patients (male/female: 43/38, age: 57 ± 17 years) who underwent kidney biopsy.

**Results:**

U-FABP4 was negatively correlated with estimated glomerular filtration rate (eGFR) (*r =* − 0.56, *P* < 0.01) and was positively correlated with age, blood pressure, triglycerides, proteinuria (*r =* 0.58, *P* < 0.01), plasma FABP4 and urinary FABP1 (U-FABP1) (*r =* 0.52, *P <* 0.01). Multivariable regression analysis showed that eGFR, proteinuria and U-FABP1 were independent predictors of U-FABP4. The level of U-FABP4, but not that of proteinuria, eGFR or U-FABP1, in minimal change nephrotic syndrome (MCNS) was significantly lower than the level in membranous nephropathy (MN) and that in diabetic nephropathy. Receiver operating characteristic curve analysis indicated that U-FABP4 level ≤ 0.78 μg/gCr predicted MCNS in patients who had nephrotic-range proteinuria with a high level of accuracy. When divided by the median value of U-FABP4 at baseline in 33 of the 81 patients who could be followed up, the yearly change (post–pre) in eGFR in the low U-FABP4 group was significantly greater than that in the high U-FABP4 group (median: 11.0 vs. -5.0 mL/min/1.73m^2^/year).

**Conclusions:**

U-FABP4 level is independently associated with proteinuria and renal dysfunction in patients with glomerular kidney disease. A low U-FABP4 level may predict MCNS in patients with nephrotic syndrome and would be a useful biomarker for differential diagnosis of MCNS and MN, which are common causes of nephrotic syndrome.

**Supplementary Information:**

**Supplementary information** accompanies this paper at 10.1186/s12882-020-02122-y.

## Background

Chronic kidney disease (CKD) and metabolic syndrome are critical issues against healthy longevity. Therefore, much attention has been paid to the associations between CKD and metabolic disorders including insulin resistance and dyslipidemia [1, 2]. Prevention and early diagnosis of CKD are important to avoid renal replacement therapy or kidney transplant, which is required in the end stage of CKD. Furthermore, even in the early stage, CKD is a risk factor for cardiovascular disease in association with metabolic diseases [3]. However, reliable biomarkers for the detection of glomerular damage at an early stage have not been developed yet [4].

The etiology needs to be determined in each case of nephrotic syndrome for selection of therapy since the response to treatment differs depending on the glomerular kidney disease. Several clinical markers, including selectivity index [5], urinary podocytes [6] and M-type phospholipase A2 receptor (PLA2R) [7], have been proposed to predict the etiology of nephrotic syndrome. It has been reported that the circulating level of anti-PLA2R antibodies is useful for diagnosis of idiopathic membranous nephropathy (MN) [7–9], but the sensitivity for diagnosis was reported to be relatively low (53%) in Japan [10]. Differential diagnosis without kidney biopsy for minimal change nephrotic syndrome (MCNS) and MN, common causes of nephrotic syndrome, is often difficult since urine findings are quite similar, i.e., urinary sediments contain little occult blood [11]. Therefore, the development of predictive markers for differential diagnosis of MCNS and MN is needed.

Fatty acid-binding proteins (FABPs), a family of intracellular lipid chaperones, are proteins of about 14–15 kDa in size that can reversibly bind to hydrophobic ligands, such as long chain fatty acids, with a high affinity and coordinate lipid responses in cells [12–15]. At least 9 different FABPs have been identified, and it has been shown that tissue-specifically expressed FABPs are secreted in various pathological conditions or leak out of cells due to cellular damage [12, 13]. Among FABPs, FABP1, known as liver-type FABP (L-FABP), is expressed in proximal tubular epithelial cells in the kidney, and this FABP isoform in urine has been reported to reflect damage of proximal tubular epithelial cells [16, 17].

FABP4, known as adipocyte FABP (A-FABP) or aP2, is expressed in both adipocytes and macrophages and contributes to the development of insulin resistance and atherosclerosis [12, 13, 18–20]. Interestingly, it has recently been reported that FABP4 is also expressed in some vascular endothelial cells [13]. In the kidney, FABP4 is expressed in endothelial cells of peritubular capillaries and veins, but not arteries or glomerular capillaries, in a normal condition [21]. We previously demonstrated that expression of FABP4 was ectopically induced by glomerular injury in cells of the glomerulus, including glomerular endothelial cells and macrophages, and that the extent of ectopic FABP4 expression was closely associated with proteinuria and renal dysfunction [22]. We also revealed in a population-based study that excretion of urinary FABP4 (U-FABP4) is associated with albuminuria and possibly predicts a yearly decline of estimated glomerular filtration rate (eGFR), suggesting that U-FABP4 would be a novel biomarker of glomerular damage [23]. However, excretion of U-FABP4 has not been examined in a study population with kidney diseases diagnosed by biopsy. Therefore, we investigated the associations between U-FABP4 level and clinical characteristics using data for a cohort of patients in whom kidney biopsies were performed.

## Methods

This study conformed to the principles outlined in the Declaration of Helsinki and was performed with the approval of the institutional ethical committee of Sapporo Medical University (number: 28–2-58) and Teine Keijinkai Hospital (number: 2017–031). The present study was separately designed from a previous study [22], in which renal expression of FABP4 in kidney biopsy samples was investigated, and did not include patients who were enrolled in the previous study. Written informed consent was obtained from all of the study subjects.

### Study subjects and clinical measurements

A total of 87 consecutive patients (male/female: 47/40) who underwent kidney biopsy in Teine Keijinkai Hospital during the period from November 2013 to April 2015 were enrolled. Samples of blood and urine were obtained before kidney biopsy during hospitalization. Six patients were excluded because volumes of stored urine samples were insufficient for analysis. After the exclusion, 81 subjects (male/female: 43/38, age: 57 ± 17 years) were finally recruited in the present study. Most of the patients returned to client hospitals after kidney biopsy for routine management, and 33 of the patients underwent follow-up examination 1 year after the biopsy in Teine Keijinkai Hospital.

eGFR was calculated by an equation for Japanese: eGFR (mL/min/1.73m^2^) = 194 × Cr^(− 1.094)^ × age^(− 0.287)^ × 0.739 (if female). Level of proteinuria was quantified as protein-to-creatinine ratio in urine (g/gCr). Concentrations of FABP4 in plasma and urine and FABP1 in urine were measured using commercially available enzyme-linked immunosorbent assay kits for FABP4 (Biovendor R&D, Modrice, Czech Republic) and FABP1 (CIMIC Co., Tokyo, Japan). The accuracy, precision and reproducibility of the kits have been described previously [17, 24]. Levels of U-FABP4 and urinary FABP1 (U-FABP1) were normalized by urine creatinine level (μg/gCr).

### Pathological diagnosis

Clinical classification and pathological diagnosis using kidney biopsy samples were determined by the WHO classification of kidney disease [25]. The indication for kidney biopsy was determined by the presence of proteinuria, hematuria or increased creatinine level. Minor glomerular abnormalities (MGA) were defined as the absence of histological abnormalities detectable by light microscope and immunofluorescence analyses in patients without nephrotic syndrome. Pathological diagnosis was corroborated by two pathologists.

### Statistical analysis

Numeric variables are expressed as means ± SD for normal distributions or medians (interquartile ranges) for skewed variables. The distribution of each parameter was tested for its normality using the Shapiro-Wilk W test, and non-normally distributed parameters were logarithmically transformed. Comparison between two groups was performed using Student’s t test for parametric parameters and the Mann-Whitney U test for nonparametric parameters. One-way analysis of variance and the Tukey-Kramer post hoc test for parametric parameters and the Kruskal-Wallis test and the Steel-Dwass post hoc test for nonparametric parameters were used for detecting significant differences in data between multiple groups. The correlation between two variables was evaluated using Pearson’s correlation coefficient. Multivariable regression analyses were performed to identify independent determinants of proteinuria, eGFR and U-FABP4 after adjustment of factors selected by stepwise regression analyses using Akaike’s Information Criterion (AIC), showing the standardized regression coefficient (β) and the percentage of variance in the object variables that the selected independent predictors explained (*R*^2^). Receiver operating characteristic curve analysis was performed to determine the inflection point at which the level of U-FABP4 provided the most sensitive prediction of MCNS in patients who had nephrotic-range proteinuria (≥ 3.5 g/gCr). The area under the curve (AUC) with the 95% confidence interval (CI) was determined, and the cut-off point was obtained by the Youden index [26]. A *p* value of less than 0.05 was considered statistically significant. All data were analyzed by using JMP 9.0.2 for Windows (SAS Institute, Cary, NC) and EZR [27].

## Results

### Clinical characteristics of the study patients

Clinical characteristics of the 81 patients who underwent kidney biopsy (mean age: 57 ± 17 years, male/female: 43/38) are shown in Table 1. The numbers of patients with IgA nephropathy /IgA vasculitis, MGA, MCNS, MN and diabetic nephropathy (DN) were 26 (32.1%), 15 (18.5%), 9 (11.1%), 12 (14.8%) and 4 (4.9%), respectively. Among the 12 patients with MN, three patients had secondary MN. Pathophysiological diagnoses of the other patients included hypertensive nephrosclerosis (*n* = 4), myeloperoxidase-antineutrophil cytoplasmic antibody-associated glomerulonephritis (*n* = 3), focal segmental glomerular sclerosis (*n* = 2), diffuse mesangial proliferative glomerulonephritis (*n =* 2), diffuse endocapillary proliferative glomerulonephritis (*n* = 1), proteinase 3-myeloperoxidase-antineutrophil cytoplasmic antibody-associated glomerulonephritis (*n =* 1), membranoproliferative glomerulonephritis (*n =* 1) and tubulointerstitial nephritis (*n =* 1). Median levels of plasma FABP4 (P-FABP4), U-FABP4 and U-FABP1 were 20.4 ng/dL, 0.55 μg/gCr and 6.78 μg/gCr, respectively (Table 1).

**Table 1:** Clinical characteristics of the patients

n	81
Age, years	57 ± 17
Male/Female	43/38
Body mass index	23.5 ± 4.2
Diagnosis ^a^	
IgA nephropathy/IgA vasculitis	23 / 3 (32.1)
MGA	15 (18.5)
MCNS	9 (11.1)
Membranous nephropathy	12 (14.8)
Diabetic nephropathy	4 (4.9)
Others	15 (18.5)
Blood pressure, mmHg	
Systolic	126 ± 21
Diastolic	73 ± 13
Laboratory data	
Creatinine, mg/dL ^b^	0.84 (0.68–1.22)
Blood urea nitrogen, mg/dL ^b^	14.0 (10.8–18.5)
eGFR, mL/min/1.73m^2^	62 ± 28
Total cholesterol, mg/dL	238 ± 101
Triglycerides, mg/dL ^b^	142 (108–215)
Fasting glucose, mg/dL	94 ± 13
Plasma FABP4, ng/mL ^b^	20.4 (13.1–33.7)
Urinary data	
Proteinuria, g/gCr ^b^	0.92 (0.25–3.77)
Hematuria ^a^	48 (59.3)
Urinary FABP4, μg/gCr ^b^	0.55 (0.21–2.34)
Urinary FABP1, μg/gCr ^b^	6.78 (1.74–20.80)

Variables are expressed as number, number (%) ^a^ means ± SD or medians (interquartile ranges) ^b^

*eGFR* estimated glomerular filtration rate, *FABP* fatty acid-binding protein, *MCNS* minimal change nephrotic syndrome, *MGA* minor glomerular abnormalities

### Associations of proteinuria, eGFR and U-FABP4 with clinical characteristics

Logarithmically transformed (log) proteinuria was negatively correlated with eGFR and was positively correlated with age, systolic and diastolic blood pressures, total cholesterol, log triglycerides, log P-FABP4, log U-FABP4 and log U-FABP1 (Table 2). Stepwise and subsequent multivariable regression analyses using age, sex and the correlated parameters revealed that total cholesterol and log U-FABP4, but not log U-FABP1, were independent predictors of proteinuria (AIC = 211, *R*^2^ = 0.57).

**Table 2:** Correlation and multivariable regression analyses for proteinuria, eGFR and urinary FABP4 (*n* = 81)

	log Proteinuria				eGFR				log Urinary FABP4			
	Correlation		Multivariable regression		Correlation		Multivariable regression		Correlation		Multivariable regression	
	r	p	β	p	r	p	β	p	r	p	β	p
Age	0.44	< 0.01	0.14	0.12	−0.63	< 0.01	−0.21	0.02	0.42	< 0.01	0.07	0.44
Sex (Male)	–	–	− 0.06	0.42	–	–	0.19	0.03	–	–	0.14	0.10
Body mass index	0.19	0.07	n.s.	–	−0.06	0.58	–	–	−0.09	0.43	–	–
Systolic blood pressure	0.38	< 0.01	n.s.	–	−0.44	< 0.01	n.s.	–	0.43	< 0.01	n.s.	–
Diastolic blood pressure	0.24	0.02	–	–	−0.23	0.38	–	–	0.24	0.04	–	–
Total cholesterol	0.48	< 0.01	0.38	< 0.01	0.02	0.83	–	–	0.07	0.53	–	–
log Triglycerides	0.41	< 0.01	–	–	−0.23	0.03	n.s.	–	0.28	0.02	n.s.	–
Fasting glucose	0.04	0.67	–	–	0.01	0.95	–	–	0.08	0.50	–	–
eGFR	−0.43	< 0.01	n.s.	–	–	–	–	–	−0.56	< 0.01	− 0.25	0.03
log Proteinuria	–	–	–	–	−0.43	< 0.01	n.s.	–	0.58	< 0.01	0.11	0.03
log Plasma FABP4	0.45	< 0.01	n.s.	–	−0.69	< 0.01	− 0.34	< 0.01	0.55	< 0.01	0.20	0.07
log Urinary FABP4	0.53	< 0.01	0.43	< 0.01	−0.56	< 0.01	−0.25	0.04	–	–	–	–
log Urinary FABP1	0.55	< 0.01	0.24	0.12	−0.48	< 0.01	−0.01	0.94	0.52	< 0.01	0.28	< 0.01
			*R*^2^ = 0.57				R^2^ = 0.51				R^2^ = 0.55	

*eGFR* estimated glomerular filtration rate, *FABP* fatty acid-binding protein

eGFR was negatively correlated with age, systolic blood pressure, log triglycerides, log proteinuria, log P-FABP4, log U-FABP4 and log U-FABP1 (Table 2). Stepwise and subsequent multivariable regression analyses using age, sex and the correlated parameters showed that age, sex, log P-FABP4 and log U-FABP4, but not log U-FABP1, were independent predictors of eGFR (AIC = 591, *R*^2^ = 0.51).

Log U-FABP4 was positively correlated with age, systolic and diastolic blood pressures, log triglycerides, log proteinuria, log P-FABP4 and log U-FABP1 and was negatively correlated with eGFR (Table 2). Stepwise and subsequent multivariable regression analyses using age, sex and the correlated parameters demonstrated that eGFR, log proteinuria and log U-FABP1 were independent predictors of U-FABP4 (AIC = 233, *R*^2^ = 0.55).

### Comparisons of renal dysfunction and urinary FABPs among glomerular kidney diseases

**Fig. 1:**
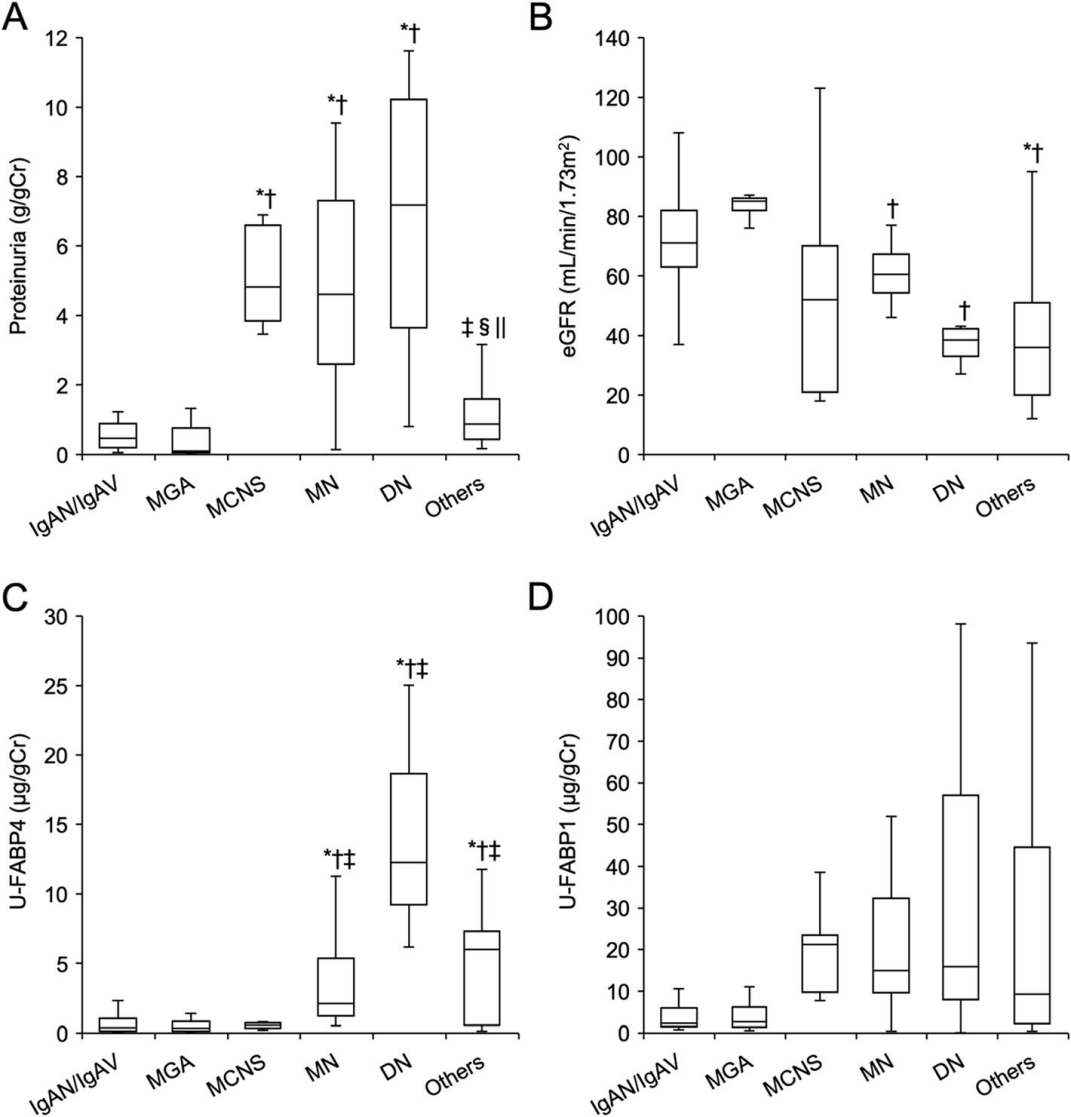
Comparisons of proteinuria, renal function and urinary FABPs among kidney diseases. **a-d**. Comparisons of levels of proteinuria (**a**), estimated glomerular filtration rate (eGFR) (**b**), urinary fatty acid-binding protein 4 (U-FABP4) (**c**) and urinary fatty acid-binding protein 1 (U-FABP1) (**d**) in patients with IgA nephropathy/IgA vasculitis (IgAN/IgAV, *n* = 26), minor glomerular abnormalities (MGA, *n* = 15), minimal change nephrotic syndrome (MCNS, *n* = 9), membranous nephropathy (MN, *n* = 12), diabetic nephropathy (DN, *n* = 4) and others (*n* = 15). *P < 0.05 vs. IgAN/IgAV, †*P <* 0.05 vs. MGA, ‡*P <* 0.05 vs. MCNS, §*P <* 0.05 vs. MN, ||*P <* 0.05 vs. DN

There were significant differences in levels of proteinuria (Fig. 1a), eGFR (Fig. 1b) and U-FABP4 (Fig. 1c), but not in levels of U-FABP1 (Fig. 1d) and P-FABP4 (Supplementary Fig. S1), among groups of IgA nephropathy/IgA vasculitis, MGA, MCNS, MN, DN and others.

When compared with groups of MCNS (*n =* 9), MN (*n* = 12) and DN (*n* = 4), which can be causes of nephrotic syndrome, there was no significant difference in levels of proteinuria (Supplementary Fig. S2A) and eGFR (Supplementary Fig. S2B) among the three groups. The level of U-FABP4 in MCNS was significantly lower than the levels in MN and DN (Supplementary Fig. S2C). There was no significant difference in the levels of U-FABP1 (Supplementary Fig. S2D).

### Prediction of MCNS in patients with nephrotic-range proteinuria

**Fig. 2:**
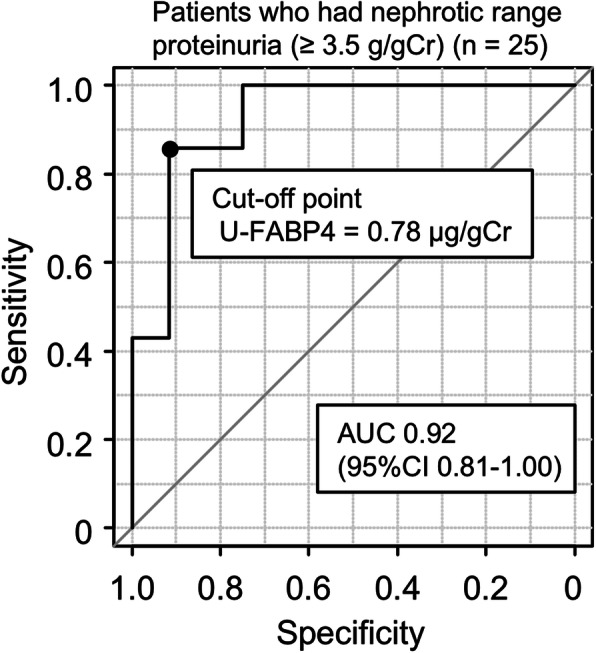
Cut-off point of U-FABP4 level to predict MCNS in patients with nephrotic-range proteinuria. Receiver operating characteristic curve analysis to determine the cut-off points of urinary fatty acid-binding protein 4 (U-FABP4) level for prediction of minimal change nephrotic syndrome (MCNS) in patients who had nephrotic-range proteinuria (≥ 3.5 g/gCr, *n* = 25)

In 25 patients who had nephrotic-range proteinuria (≥ 3.5 g/gCr), including MCNS (*n* = 9), MN (*n* = 7), DN (*n* = 3), IgA vasculitis (*n =* 3), focal segmental glomerular sclerosis (*n* = 2) and membranoproliferative glomerulonephritis (*n* = 1), the AUC of receiver operating characteristic curve analysis for U-FABP4 level to predict MCNS was 0.92, and the cut-off point of U-FABP4 level by the Youden index was 0.78 μg/gCr (Fig. 2), indicating that a level of U-FABP4 ≤ 0.78 μg/gCr predicts MCNS.

### Yearly changes in eGFR in the low and high groups of urinary FABPs

**Fig. 3:**
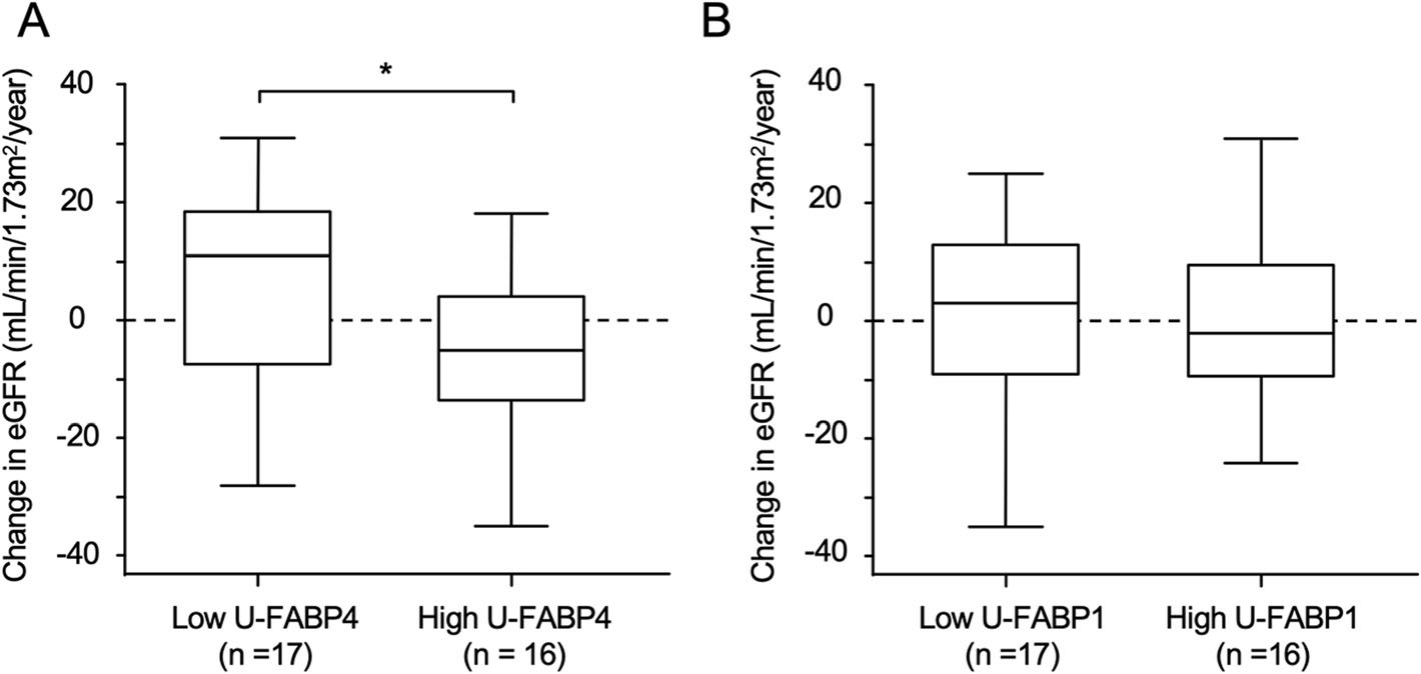
Yearly declines in eGFR in low and high groups of urinary FABPs. **a**, **b**. Yearly changes (post - pre) in estimated glomerular filtration rate (eGFR) in patients (*n* = 33) divided by median values of urinary fatty acid-binding protein 4 (U-FABP4) (0.72 μg/gCr) (**a**) and urinary fatty acid-binding protein 1 (U-FABP1) (6.77 μg/gCr) (**b**). *P < 0.05

Follow-up examination after 1 year in our institute (Teine Keijinkai Hospital) was performed in 33 of the 81 patients. The patients were divided into two groups by the median value of U-FABP4 or U-FABP1 at baseline: low and high groups. The yearly change in eGFR, defined as the value at one-year follow-up minus the baseline value, in the low U-FABP4 group was significantly greater than that in the high U-FABP4 group (median: 11.0 vs. -5.0 mL/min/1.73m^2^/year) (Fig. 3a). There was no significant difference in the yearly change in eGFR between the low and high U-FABP1 groups (Fig. 3b).

## Discussion

The present study demonstrated for the first time that U-FABP4 level has a significant association with renal dysfunction and prognosis in patients with biopsy-based diagnosis of kidney diseases. The level of U-FABP4 was independently associated with levels of proteinuria and eGFR in patients with kidney disease, and the association of U-FABP4 with proteinuria or eGFR was independent of circulating FABP4 and U-FABP1, which reflects damage of proximal tubular epithelial cells [16, 17]. The level of U-FABP4 in MCNS was significantly lower than that in MN and that in DN, and a low U-FABP4 level predicted MCNS in patients who had nephrotic-range proteinuria (≥ 3.5 g/gCr) with a high level of accuracy (AUC: 0.92). Therefore, the level of U-FABP4 would be a useful biomarker for differential diagnosis of MCNS and MN, which are common causes of nephrotic syndrome.

It has been reported that the circulating level of anti-PLA2R antibodies is useful for diagnosis of idiopathic MN [7–9]. However, the sensitivity of the circulating level of anti-PLA2R antibodies for diagnosis of idiopathic MN was reported to be relatively low (53%) in Japan [10] unlike in other populations in a meta-analysis (78%) [9]. In addition, both idiopathic MN and secondary MN were included in the present study. Therefore, the level of U-FABP4 might be more useful than the level of anti-PLA2R antibodies for differential diagnosis of MCNS and both idiopathic and secondary types of MN. Further studies using a large number of patients are needed to clarify the significance of U-FABP4 as a useful biomarker for differential diagnosis of MCNS and MN by comparisons of several clinical markers such as selectivity index [5], urinary podocytes [6] and PLA2R [7].

FABP4 is not expressed in vascular endothelial cells of the artery and glomerulus in a normal condition [21]. It has been reported that ectopic FABP4 expression is induced by cellular senescence and arterial vascular injury in vascular endothelial cells [13, 28, 29]. We previously showed that protein and mRNA levels of FABP4 were ectopically expressed in glomerular endothelial cells by glomerular injury [22]. The semiquantitative area of FABP4 expression in the glomerulus was positively correlated with proteinuria and was negatively correlated with the yearly change in eGFR [22]. FABP4 was thought to be a non-secreted protein since it does not have an apparent signal peptide in the amino acid sequence [12], but it has recently been shown that FABP4 is secreted from adipocytes in association with lipolysis via a non-classical pathway [30]. We recently demonstrated that ectopically expressed FABP4 is secreted from injured vascular endothelial cells, contributing to the development of neointima formation after vascular injury [29]. Similar mechanisms of local expression and secretion of FABP4 may underlie the pathogenesis in glomerular endothelial cells. Since glomerular endothelial cells have been proposed as a target for therapy to improve outcomes of kidney diseases [31], manipulation of ectopically expressed FABP4 in the glomerulus and/or a mechanism underlying urinary excretion of FABP4 could be a strategy for prevention of glomerular kidney disease.

FABP4 can pass through glomerular filtration-sized and negatively charged barriers because of its small molecular weight (about 15 kDa) [12] and because of the net positive surface electrostatic potential across the portal region of collisional FABPs [32, 33]. Circulating FABP4 has been reported to be processed by the kidney and reabsorbed through megalin, an endocytic receptor expressed in proximal tubule epithelial cells [34]. It has also been shown that U-FABP4 is increased before an increase in serum creatinine, clusterin or cystatin C in a drug-induced glomerulonephritis model with N-phenylanthranilic acid and puromycin in in vivo and in vitro studies [35]. Newly expressed and secreted FABP4 in the glomerulus may pass through glomerular filtration barriers, and an excess of FABP4, which exceeds the permissible amount of reabsorption in proximal tubular cells, is excreted into urine, resulting in reflection of the extent of glomerular injury.

Since levels of P-FABP4 were comparable in MCNS and MN in patients with nephrotic syndrome (Supplementary Fig. S1), the difference in U-FABP4 between the two groups would be attributable to new expression of FABP4 in the kidney. Crosstalk between glomerular endothelial cells and podocytes in glomerular injury has recently been highlighted [36, 37], and an association between glomerular endothelial cells and podocytes mediated by vascular endothelial growth factor (VEGF) has been suggested in patients with MN [38–41]. It has also been shown that ectopic expression of FABP4 in endothelial cells is induced by a VEGF-VEGF-receptor 2 pathway [21]. U-FABP4 derived from glomerular endothelial cells, which is possibly induced by VEGF, may affect podocytes, leading to the development of proteinuria.

It has been reported that U-FABP1 level is a marker of tubulointerstitial injury [16, 17] and progression of renal dysfunction in CKD patients [42, 43]. In the present study, there was no significant difference in the yearly change in eGFR between the low and high U-FABP1 groups (Fig. 3b), probably due to the small number of patients who could be followed up (*n* = 33). On the other hand, the yearly change in eGFR in the low U-FABP4 group was significantly greater than that in the high U-FABP4 group (Fig. 3a). The median value of yearly change in eGFR in the low U-FABP4 group was 11.0 mL/min/1.73m^2^/year, whereas that in the high U-FABP4 group was − 5.0 mL/min/1.73m^2^/year, suggesting deterioration of renal function. The low U-FABP4 group may have included kidney diseases with a high rate of response to treatment such as MCNS. Thus, the findings regarding the relationship between U-FABP4 and prognosis of eGFR are preliminary findings.

The present study had several limitations. First, since the recruited subjects were only Japanese, the results of this study may not be directly extrapolated to other ethnic groups. Second, the possibility of a selection bias in the samples cannot be excluded since the study was conducted in a single facility. Third, the number of patients enrolled in the present study was small. A multicenter study with a large number of patients is needed for confirming the associations of U-FABP4 with clinical characteristics in patients with various kidney diseases. Especially, the number of patients with DN was small among patients with nephrotic-range proteinuria. Therefore, additional large-scale studies to investigate the association between U-FABP4 and DN are needed in the future. Finally, some of the drugs used for treatment, including steroids, immunosuppressive agents and renin-angiotensin system inhibitors, might have affected data for the change in eGFR as unadjusted confounding factors.

## Conclusions

U-FABP4 level is independently associated with proteinuria and renal dysfunction in patients with glomerular kidney disease. A low U-FABP4 level may predict MCNS in patients with nephrotic syndrome and may be a biomarker that is useful for differential diagnosis of MCNS and MN, which are common causes of nephrotic syndrome.

## Supplementary Information

**Additional file 1: Figure S1.** Comparisons of plasma FABP4 among kidney diseases. **Figure S2.** Comparisons of proteinuria, renal function and urinary FABPs among kidney diseases which cause nephrotic syndrome.

## Data Availability

The datasets used and analyzed during the current study are available from the corresponding author on reasonable request.

## Abbreviations

AUC: Area under the curve
CKD: Chronic kidney disease
CI: Confidence interval
DN: Diabetic nephropathy
eGFR: Estimated glomerular filtration rate
FABP: Fatty acid-binding protein
MCNS: Minimal change nephrotic syndrome
MGA: Minor glomerular abnormalities
MN: Membranous nephropathy
PLA2R: M-type phospholipase A2 receptor
P-FABP4: Plasma fatty acid-binding protein 4
U-FABP1: Urinary fatty acid-binding protein 1
U-FABP4: Urinary fatty acid-binding protein 4
