# Supplementary material for: Significance of urinary fatty acid-binding protein 4 level as a possible biomarker for the identification of minimal change disease in patents with nephrotic-range proteinuria
Source: BMC Nephrol. 2020 Nov 3;21:459. doi: 10.1186/s12882-020-02122-y (PMC7640424; doi:10.1186/s12882-020-02122-y)
Supplement: Supplementary file 1 — Additional file 1: Figure S1. Comparisons of plasma FABP4 among kidney diseases. Figure S2. Comparisons of proteinuria, renal function and urinary FABPs among kidney diseases which cause nephrotic syndrome. [file 12882_2020_2122_MOESM1_ESM.pdf]

Figure S1

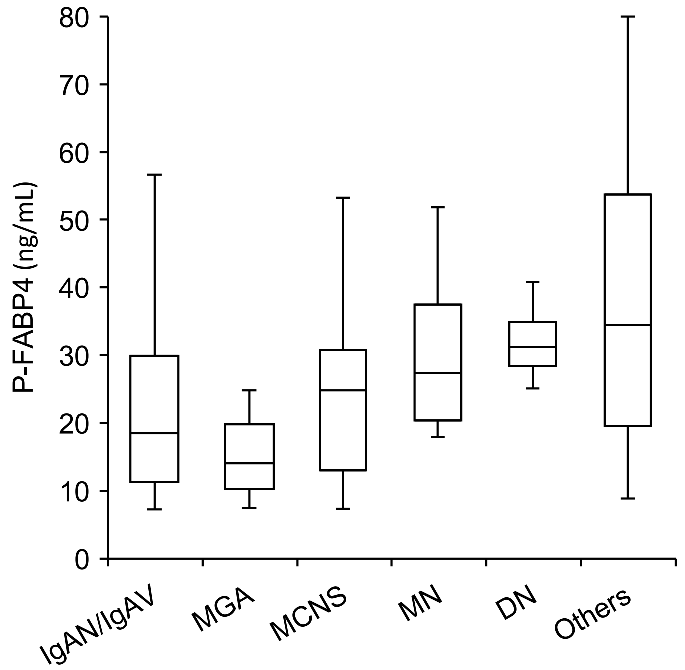

Supplementary Figure S1. Comparisons of plasma FABP4 among kidney diseases.

Comparisons of levels of plasma fatty acid-binding protein 4 (P-FABP4) in patients with IgA nephropathy/IgA vasculitis (IgAN/IgAV, n = 26), minor glomerular abnormalities (MGA, n = 15), minimal change nephrotic syndrome (MCNS, n = 9), membranous nephropathy (MN, n = 12), diabetic nephropathy (DN, n = 4) and others (n = 15).

Figure S2

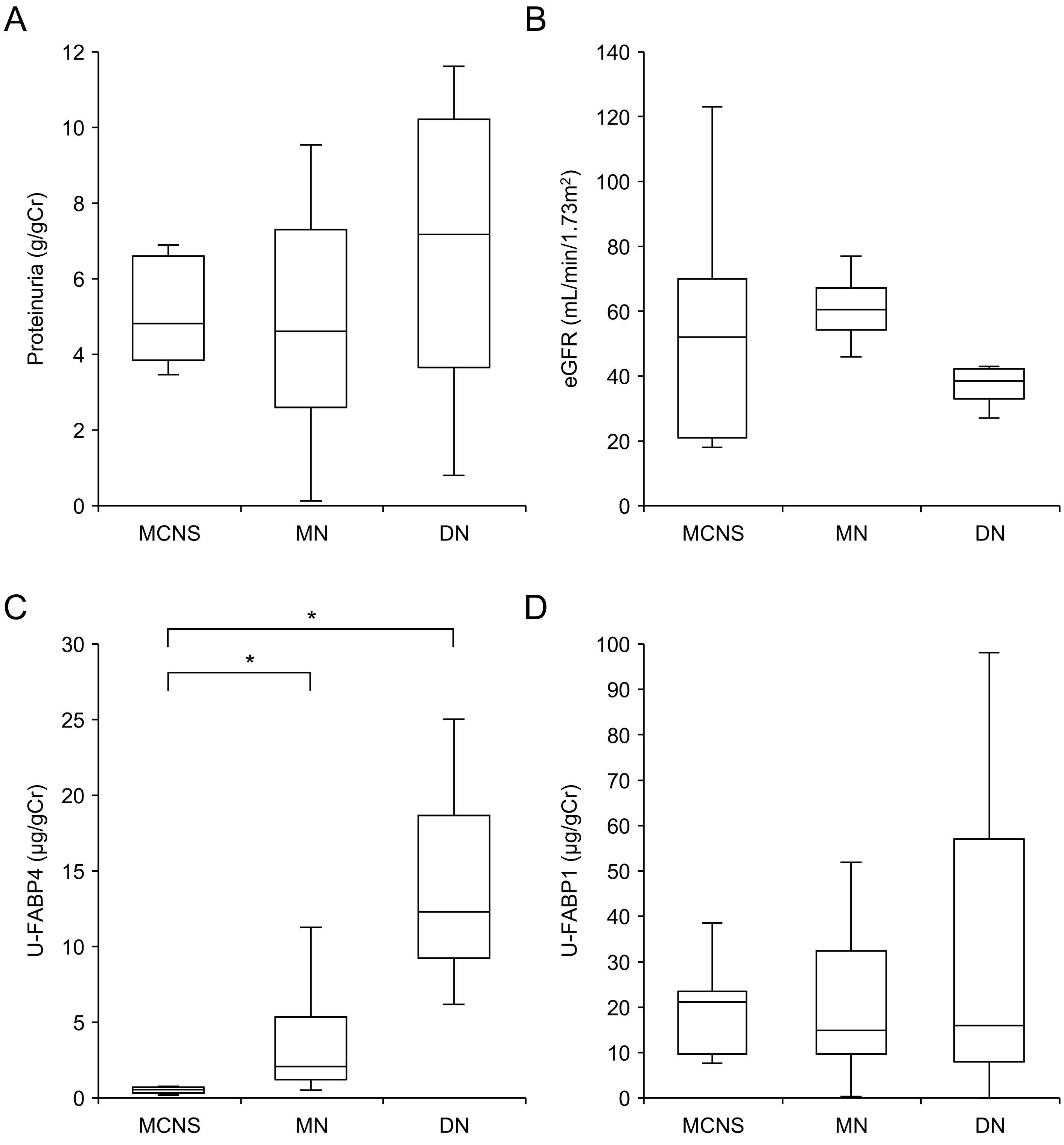

Supplementary Figure S2. Comparisons of proteinuria, renal function and urinary FABPs among kidney diseases which cause nephrotic syndrome.

A-D. Comparisons of levels of proteinuria (A), estimated glomerular filtration rate (eGFR) (B), urinary fatty acid-binding protein 4 (U-FABP4) (C) and urinary fatty acid-binding protein 1 (U-FABP1) (D) in patients with minimal change nephrotic syndrome (MCNS, n = 9), membranous nephropathy (MN, n = 12) and diabetic nephropathy (DN, n = 4). *P < 0.05.
